# Paradoxical bronchospasm: a rare adverse effect of fenoterol use

**DOI:** 10.1002/rcr2.698

**Published:** 2021-03-17

**Authors:** Ching‐Han Lai, Xin‐Min Liao

**Affiliations:** ^1^ Division of Pulmonary Medicine, Department of Internal Medicine National Cheng Kung University Hospital Tainan Taiwan

**Keywords:** Asthma, beta‐agonist inhalers, paradoxical bronchospasm

## Abstract

Paradoxical bronchospasm refers to the constriction of the airways after treatment with a sympathomimetic bronchodilator. Theoretically, bronchodilators, such as beta‐agonist inhalers, act to ease asthma symptoms by relaxing the muscles surrounding the walls of the bronchial tubes, which relieve bronchial constriction. However, in rare instances, some patients develop respiratory distress or even respiratory failure after inhaled bronchodilator use, although the exact mechanism for this adverse effect is unknown. We report a male, with a known asthma history diagnosed for more than one decade, receiving fenoterol (Berotec®) for wheezing control and the worsening of his clinical condition immediately after bronchodilator administration.

## Graphical abstract

In this brief study, we presented a case with a rarely seen adverse effect after fenoterol inhalation. To the best of our knowledge, this is the first reported case of fenoterol‐induced paradoxical bronchospasm. It is crucial that clinicians are aware of this unexpected adverse event to provide prompt monitoring of patients to improve outcomes.

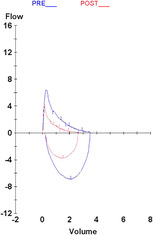

## Introduction

Paradoxical bronchospasm means a constriction of the airways after treatment with a sympathomimetic bronchodilator. Selective beta 2‐agonist inhalers are the most potent bronchodilators currently approved for clinical use in asthma and obstructive lung disease. Theoretically, bronchodilators act to ease symptoms by relaxing the muscles surrounding the walls of the bronchial tubes, which relieve bronchial constriction [1, 2, 3]. However, in rare cases, some patients develop aggravating ordinary respiratory discomforts, even respiratory distress or acute respiratory failure events, after bronchodilator use. Be that as it may, the exact mechanism for this adverse effect remains unclear. Here, we report a man, with a known asthma history diagnosed for over one decade, receiving fenoterol (Berotec®, Boehringer‐ingelheim, Taiwan) for wheezing control and the worsening of his clinical condition, including being intubated twice due to acute respiratory failure, which occurred less than 5 min immediately after one puff of fenoterol (Berotec) inhalation.

## Case Report

The 50‐year‐old gentlemen had a previous history of type 2 diabetes mellitus (DM) and bronchial asthma, both of which had been diagnosed for over one decade. Drug allergy to penicillin was also documented in his previous medical records. In mid‐June of 2013, he had one common cold event took one puff of fenoterol (Berotec) due to dyspnoea. However, his dyspnoea worsened, and his wheezing sound became more prominent after fenoterol (Berotec) inhalation. He was sent to the emergency department of a regional hospital in Kaohsiung for treatment. The symptoms were relieved after ipratropium and terbutaline inhalation, and he was discharged via the emergency room on the same day. One week later in late June, he suffered another wheezing attack and had one puff of fenoterol (Berotec) inhalation for symptoms relief at home; however, his dyspnoea worsened. He was sent to the hospital again, and this time, received intubation due to acute respiratory failure. The patient was extubated and discharged in early July after a good recovery. A similar event also happened once in early August, but he did not receive invasive mechanical ventilation therapy because it presented as a milder clinical condition. The patient reported that he had had one puff of fenoterol (Berotec) inhalation before wheezing worsened.

He was admitted to one regional hospital in Kaohsiung in early September 2013 due to acute respiratory failure. Common cold‐associated symptoms, such as productive cough and dyspnoea, were noted for one week before he was sent to the hospital. On the same day, he was admitted to the hospital, and dyspnoea with a wheezing breathing sound developed in the morning. He received one puff inhalation of fenoterol (Berotec) at home, yet dyspnoea was aggravated instead of being relieved. He was sent to the hospital by ambulance, and intubation was performed immediately after his arrival at the emergency room due to his poor respiratory pattern. After 30 min of intubation, he was extubated as his clinical condition improved. Upon going to our pulmonology outpatient department for further evaluation, the chest X‐ray showed no obvious lung lesion. The blood eosinophil count was 354/μL and the IgE level was 564 IU/mL. We noticed he had a prior intubation episode mentioned above due to acute respiratory failure in late June 2013. Coincidentally, one puff of fenoterol (Berotec) was inhaled by the gentleman before each intubation episode, according to his statements.

Tracing back his medical records, his occupation was an office worker in a factory (screw manufacturer) and was rarely exposed to the raw materials. He had been diagnosed as having DM for more than two decades and was under regular follow‐ups at the outpatient department of family medicine at National Cheng Kung University Hospital (NCKUH). He took metformin, vildagliptin, and glimepiride for DM control. As for his asthma history, he was diagnosed as having asthma when he was 40 years old at a regional hospital in Kaohsiung. However, there is no known asthma history for any of his family members. Fluticasone/salmeterol has been prescribed as the controller since the asthma was diagnosed and it was well tolerated. The asthma was under good control since hardly did he have acute exacerbation, as he reported. Although fenoterol (Berotec) had been prescribed for wheezing control in the prior decade, not until the June 2013 event did he receive inhaled fenoterol (Berotec) therapy for a wheezing attack.

**Figure 1:**
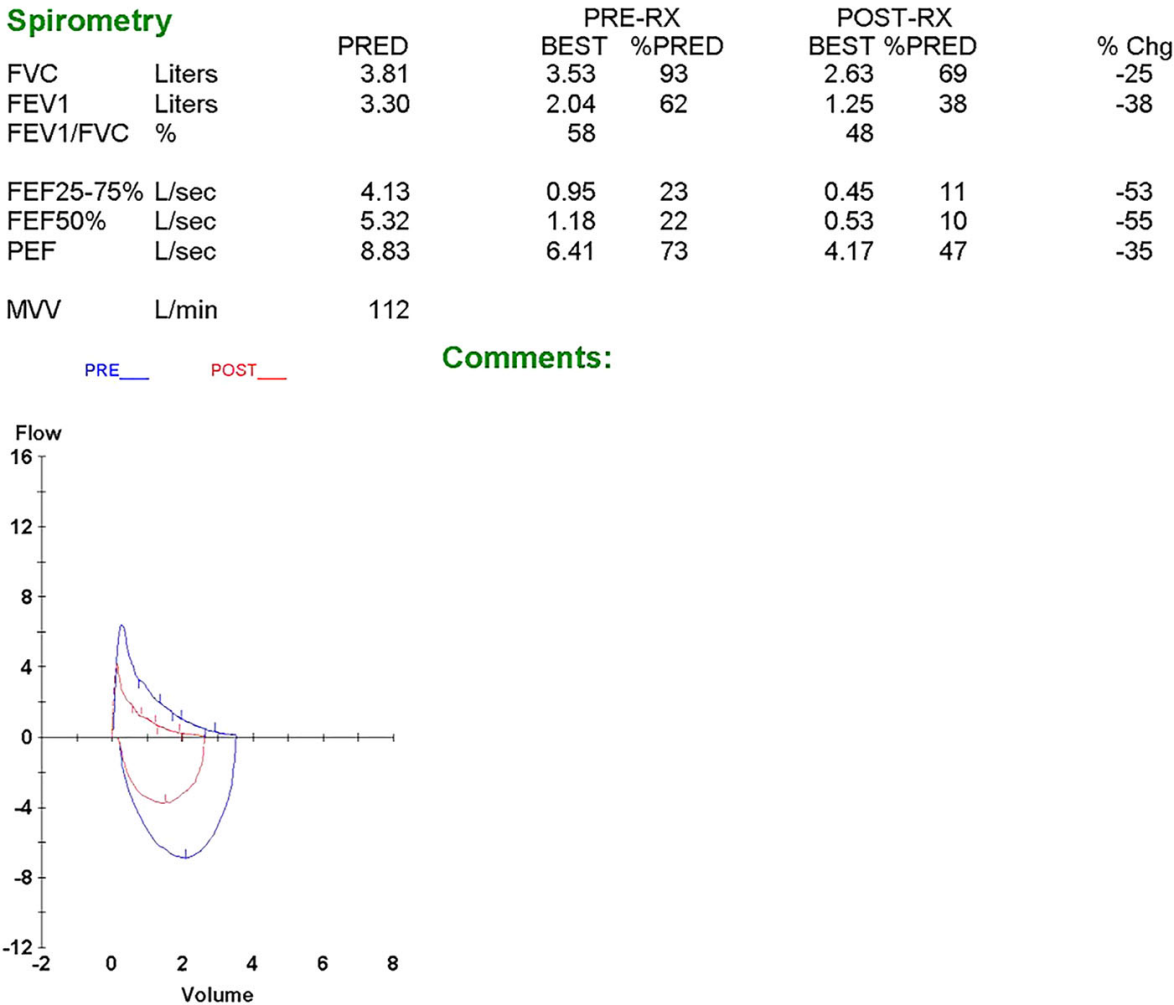
Spirometry of bronchodilator (fenoterol) test is demonstrated. The blue circuit is the pre‐bronchodilator spirometry and the red circuit is the post‐bronchodilator spirometry. Chg, change; FEF, forced expiratory flow; FEV_1_, forced expiratory volume in 1 sec; FVC, forced vital capacity; MVV, maximal voluntary ventilation; PEF, peak expiratory flow; PRE‐RX, pre‐prescription; PRED, prediction; POST‐RX, post‐prescription.

Considering his clinical presentations, paradoxical bronchospasm was suspected. We reported this case and made bronchodilator adjustments at our pulmonology clinic later. After discussing with the patient, bronchodilator (fenoterol) test was performed in mid‐September of 2013 under close monitoring and informed consent, which reported negative impact on both forced expiratory volume in 1 sec (FEV_1_) and forced vital capacity (FVC) (Fig. 1).

He previously had fluticasone/salmeterol (Seretide® Accuhaler, GSK, Taiwan) as the controller for asthma treatment. The controller was switched to fluticasone/vilanterol (Relvar 92/22®, GSK, Taiwan), and umeclidinium (Incruse®, GSK, Taiwan) was added consecutively. Symbicort®, Astrazeneca, Taiwan, as the reliever, was administered once and no paradoxical bronchospasm event developed. He is now under regular outpatient department follow‐up at NCKUH.

## Discussion

Paradoxical bronchospasm is defined as the sudden onset of an unanticipated contraction of smooth muscle in the walls of the bronchi occurring soon after the administration of an aerosolized bronchodilator. Rare, but not isolated, transient reductions of FEV_1_ have been observed in previously healthy subjects after inhalation in two phase I trials [4].

Several mechanisms have been proposed to account for the paradoxical bronchoconstriction observed with beta 2‐agonist metered‐dose inhaler (MDI), including an IgE‐mediated reaction to excipients in the MDI (e.g. soya bean lecithin) [5], as well as secondary irritation to propellants, preservatives, or turbulence of airflow due to inappropriate inhaler technique [6]. Hypotheses have been proposed to account for the paradoxical bronchospasm that has occurred with nebulized beta 2‐agonists, including bronchial irritation caused by the hyper‐/hypo‐osmolality and acidity [7] of the solution as well as preservatives (e.g. sodium metabisulphite, benzalkonium chloride, and ethylenediaminetetraacetic acid (EDTA)) [8, 9] (Table 1). Zhong et al. demonstrated a case that did not induce paradoxical bronchospasm from the same inhaler by removing the preservatives [11]. Moreover, the same major content with different excipients or preservatives led to a different impact on FEV_1_ [10, 13]. Several previous retrospective studies have revealed that the statistical incidence of paradoxical bronchospasm was <1%.

**Table 1:** Cases of paradoxical bronchospasm from 1986 to 2018.

Reference	Number of cases/Age and Sex or details	Diagnosis	Medication	Possible aetiology	Final medication
Magee and Pittman, 2018 [10]	1/25 M	Asthma	Albuterol	Excipient	Ipratropium
George et al., 2017 [8]	1/17 F	Asthma	Albuterol	BAC	Levabuterol/BAC free
Zhong et al., 2014 [11]	1/68 F	Asthma	Salbutamol	Enantiomer	Not mentioned
Broski and Amundson, 2008 [12]	1/36 M	Asthma	Levalbuterol	At least not HFA	Salmeterol and fluticasone
Spooner and Olin, 2005 [13]	1/92 M	COPD	Albuterol	Not mentioned	Not mentioned
Mutlu et al., 2000 [9]	1/22 F	Asthma	Albuterol/metaproterenol	EDTA	ICS, theophylline
Facchini et al., 1996 [18]	1/age not reported F	Asthma	Not mentioned	Soya‐derived excipients	Not mentioned
Jorup et al., 2014 [4]	5/3 COPD and 2 healthy	COPD/healthy FEV_1_↓	LAMA AZD9164	Not mentioned	Not mentioned
O'Callaghan et al., 1986 [7]	17/Infants	Wheezing/asthma	Salbutamol	High osmolality and acidity	Not mentioned

BAC, benzalkonium chloride; COPD, chronic obstructive pulmonary disease; EDTA, ethylenediaminetetraacetic acid; FEV_1_, forced expiratory volume in 1 sec; HFA, hydrofluoroalkane; ICS, inhaled corticosteroid; LAMA, long‐acting muscarinic antagonist.

We reviewed the literature concerning paradoxical bronchospasm, the published case reports of which are shown in Table 1 and the prevalence of paradoxical bronchospasm for SABA, LABA, and long‐acting muscarinic antagonist (LAMA) are listed in Table 2. We reviewed the literature on the PubMed®, using “paradoxical bronchospasm/bronchoconstriction” as the key word and 63 results were searched. The results narrowed down to 18 if we add “case report” as the key word. The literature was limited to studies that have open access to the full text and in English (or English translations), which resulted in exclusion of 4 studies. The time of publication is not limited. Eight studies were excluded because the bronchospasm is not bronchodilator‐induced. The phenomenon was more prevalent in asthma subjects than in those with chronic obstructive pulmonary disease (COPD), indicating that asthma sufferers might be more susceptible to the development of paradoxical bronchospasm. Within the six reported paradoxical bronchospasm cases, four patients were using albuterol, while the other two cases were using salbutamol. The speculated aetiology of paradoxical bronchospasm varied, including excipient, preservatives (benzalkonium chloride), and enantiomer.

**Table 2:** Prevalence of paradoxical bronchospasm for SABA, LABA, and LAMA.

Medication	Prevalence (%)	Reference
Albuterol/salbutamol	1–8	14, 15
Salmeterol (Seretide Evohaler)	<0.01	16
Tiotropium	<1	17

LABA, long‐acting beta2 agonist; LAMA, long‐acting muscarinic antagonist; SABA, short‐acting beta2 agonist.

In this brief study, we presented a case with a rarely seen adverse effect after fenoterol inhalation. Paradoxical bronchospasm is one of the adverse effect listed on the package insert of fenoterol (Berotec®) but it is rarely reported. Bronchodilator test using fenoterol as the bronchodilator was performed on this patient and showed reductions of FEV_1_ and FVC, compatible with the fenoterol‐induced paradoxical bronchospasm. It is crucial that clinicians are aware of this unexpected adverse event to provide prompt monitoring of patients to improve outcomes.

### Disclosure Statement

Appropriate written informed consent was obtained for publication of this case report and accompanying images.
